# Correction of unexpected distributions of P values from analysis of whole genome arrays by rectifying violation of statistical assumptions

**DOI:** 10.1186/1471-2164-14-161

**Published:** 2013-03-11

**Authors:** Sheila J Barton, Sarah R Crozier, Karen A Lillycrop, Keith M Godfrey, Hazel M Inskip

**Affiliations:** 1 MRC Lifecourse Epidemiology Unit, University of Southampton, Southampton, UK; 2 NIHR Southampton Biomedical Research Centre, University of Southampton and University Hospital Southampton NHS Foundation Trust, Southampton, UK; 3 Human Development and Health Academic Unit, University of Southampton, Southampton, UK; 4 School of Biological Sciences, University of Southampton, Southampton, UK

**Keywords:** P values, Distributions, Statistical analysis, Statistical assumptions, Whole genome methylation promoter arrays, Epigenome

## Abstract

**Background:**

Statistical analysis of genome-wide microarrays can result in many thousands of identical statistical tests being performed as each probe is tested for an association with a phenotype of interest. If there were no association between any of the probes and the phenotype, the distribution of P values obtained from statistical tests would resemble a Uniform distribution. If a selection of probes were significantly associated with the phenotype we would expect to observe P values for these probes of less than the designated significance level, alpha, resulting in more P values of less than alpha than expected by chance.

**Results:**

In data from a whole genome methylation promoter array we unexpectedly observed P value distributions where there were fewer P values less than alpha than would be expected by chance. Our data suggest that a possible reason for this is a violation of the statistical assumptions required for these tests arising from heteroskedasticity. A simple but statistically sound remedy (a heteroskedasticity–consistent covariance matrix estimator to calculate standard errors of regression coefficients that are robust to heteroskedasticity) rectified this violation and resulted in meaningful P value distributions.

**Conclusions:**

The statistical analysis of ‘omics data requires careful handling, especially in the choice of statistical test. To obtain meaningful results it is essential that the assumptions behind these tests are carefully examined and any violations rectified where possible, or a more appropriate statistical test chosen.

## Background

In the last 10 years microarrays have become a fundamental tool in biological research laboratories throughout the world. The recent growth in interest of applying this technology to studying a different aspect of the genome, namely the epigenome, requires that researchers have a good understanding of the biological processes underlying the new array technologies and their applications as well as the statistical issues involved when analysing the resulting data. Whilst there are many publications exploring the biology of DNA methylation and the epigenome, and a large number of articles describing the development of approaches for studying DNA methylation, there are few articles that address aspects of the analytic issues involved in these new technologies.

In genome-wide epigenetic analyses a small number of samples are typically submitted to a whole genome methylation array due to cost and sample availability constraints; these arrays can generate in excess of 500,000 data points (probes) for each sample. If the samples are chosen to be extremes of available phenotype, statistical tests appropriate for two groups, such as t-tests or Mann–Whitney U tests, can be used on the methylation results for each probe. If the samples are chosen across the full range of phenotype values, techniques such as linear regression can be used to test the association of phenotype with methylation results. Both of these approaches lead to a large number of statistical tests based on a small number of subjects and statistical techniques have been designed to accommodate this [1,2].

If there were no association between any of the probes measuring methylation of the various CpG sites in the array and the phenotype, the distribution of P values from the statistical tests used would approximate a Uniform distribution. A Uniform distribution can be visualised as a histogram where each bar is equal in height, as each possible P value is equally likely to occur. For example if you consider putting each P value (with possible values ranging from 0 to 1) into bins of width 0.05, as each P value is equally likely to occur the same number of P values are likely to be put into each bin and would therefore produce bars of equal height when plotted on a histogram. If however some probes are significantly associated with the phenotype of interest we would expect an excess of P values less than alpha (typically alpha is 0.05 or less) due to the association; the P value distribution would no longer be a Uniform distribution and a histogram would show longer bars representing P values less than alpha resulting from an excess of probes significantly associated with the phenotype.

In exploratory analyses using a whole genome methylation promoter array we observed a P value distribution for some outcomes where fewer P values less than alpha were observed than would be expected by chance. We hypothesised that this could be due to some of the assumptions of the statistical technique used being violated and sought a solution to this problem. Our solution is a new approach which differs from the published literature [3] and is much easier to implement using routines already contained in a variety of statistical software packages including R [4].

## Results and discussion

Methylation at the various CpG sites across the genome was measured using the log of the ratio of methyl to input signal (see Materials and methods), hereafter referred to as the log ratio value. The association of log ratio values for approximately 240,000 probes designed to measure methylation of CpGs situated on the promoter regions of genes on chromosomes 1 to 10, with a continuous neuro-cognitive outcome were obtained using linear regression controlling for gender. The distribution of P values obtained for the regression coefficients is shown in Figure 1; this shows that there are more P values ≤ 0.05 than would be expected by chance and thus suggests that some probes are significantly associated with the neuro-cognitive outcome.

**Figure 1:**
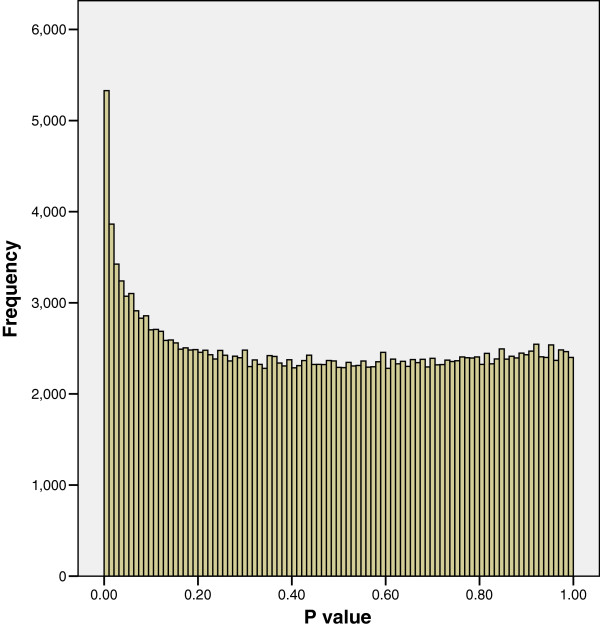
**The distribution of P values for regression coefficients with a neuro-cognitive outcome.** The association of 237,152 probe log ratio values with a continuous neuro-cognitive outcome were obtained using linear regression controlling for gender. Figure 1 shows that there are more P values ≤ 0.05 than would be expected by chance and thus suggests that some probes are significantly associated with the neuro-cognitive outcome.

A simulation exercise was undertaken by randomly permuting the outcome values across all participants with respect to the probe log ratio values for each regression and recalculating the regression coefficients and corresponding P values. Figure 2 shows the P value distribution obtained; as expected there are no more P values ≤ 0.05 than would be expected by chance, resulting in a distribution similar to a Uniform distribution.

**Figure 2:**
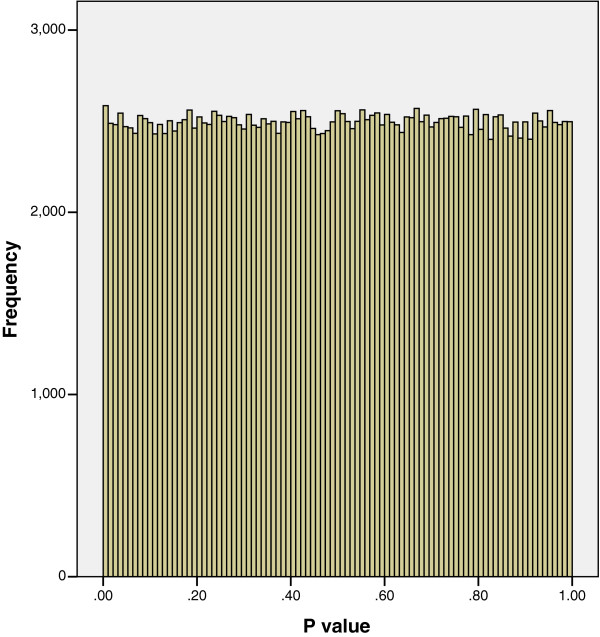
**The distribution of P values after a simulation exercise using permutation of outcome values.** Figure 2 shows there are no more P values ≤ 0.05 than would be expected by chance, resulting in a distribution similar to a Uniform distribution.

When the associations between the probe log ratio values and a continuous body composition outcome were assessed using linear regression, the distribution of P values shown in Figure 3 was obtained; fewer P values ≤ 0.05 were obtained than would be expected by chance. The distribution of P values in Figure 3 is unexpected; if there were no association between any of the probes and the outcome, each P value would be equally likely to occur and a distribution similar to Figure 2 would be obtained. In the few occasions in published literature that a distribution similar to Figure 3 has been reported [3,5] this was thought to be due to technical issues concerned with imperfect normalisation between plates or cross-hybridisation in the array. Neither of these potential explanations is likely for our results, as one outcome produced the expected distribution of P values (see Figure 1) and a second outcome analysed in relation to the same probe log ratio values produced the unexpected P value distribution in Figure 3. The distribution of P values obtained from regressions based on chromosomes 11 to 22, X and Y with the neuro-cognitive and body composition outcomes were very similar to Figures 1 and 3 respectively. Therefore in the interests of clarity and brevity we have only reported on the analysis of chromosomes 1 to 10.

**Figure 3:**
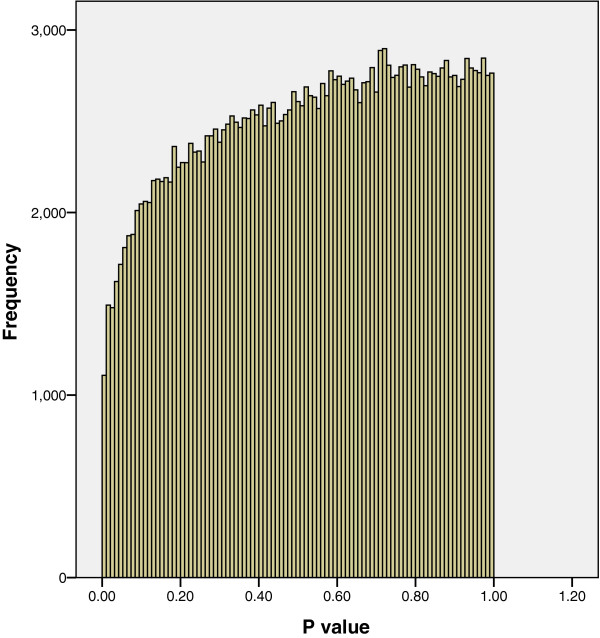
**The distribution of P values for regression coefficients with a body composition outcome.** Figure 3 shows that fewer P values ≤ 0.05 were obtained than would be expected by chance.

In response to an anonymous reviewer we considered whether correlation of log ratio values between probes located in the promoter region of the same gene could be affecting the distribution of P values. The median number of probes in the promoter region of the genes was found to be 16, with a lower quartile of 10 and an upper quartile of 23. The 237,152 probes are located in or around 8,782 genes (around 4% the number of probes) on chromosomes 1 to 10. We therefore took a random sample of 4% of the P values for each outcome so that adjacent probes in the same gene are unlikely to be selected. As we observed a distribution of P values for these 4% datasets similar to Figure 1 for the neuro-cognitive outcome and similar to Figure 3 for the body composition outcome, we conclude that correlation between probes located in the same promoter region is unlikely to affect the shape of the P value distribution.

We hypothesised that the reason that fewer P values ≤ 0.05 were being obtained than would be expected by chance could be due to violation of assumptions for the statistical test or procedure used (in this case linear regression). Therefore a thorough investigation was carried out to check regression assumptions for a selection of probes. Scatter plots were used to check the assumption of a linear relationship between outcome and predictor variables. Plots of the residuals from the regressions (the difference between the value of the outcome as predicted by the regression equation and the actual outcome value) were checked for obvious deviations from a Normal distribution using a Probability-Probability (P-P) plot and for heteroskedasticity (non-constant variance of residuals i.e. variance of residuals increasing or decreasing with size of predicted value). For the probes investigated there was no obvious non-linearity between probe log ratio value and outcome, and the P-P plots did not show extreme deviation from normality. However when the residuals were plotted against predicted values for a selection of probes several of the plots showed evidence for heteroskedasticity; Figure 4 shows one of these plots, in which the regression residuals increased with increasing predicted values (the points on the plot spread out in a fan shape), reflecting heteroskedasticity.

**Figure 4:**
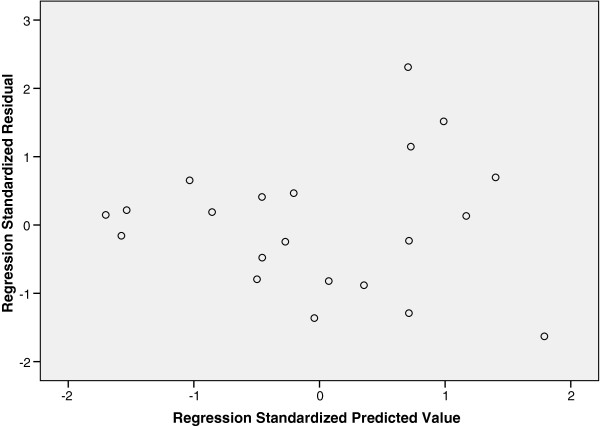
**Regression residuals for a body composition outcome plotted against values predicted by the regression.** Figure 4 shows the regression residuals increasing with predicted value (the points on the plot spread out in a fan shape), indicating heteroskedasticity.

It was therefore decided to test the residuals from all 237,152 regressions with the body composition outcome for heteroskedasticity using the Cook-Weisberg test, a standard statistical test [6] implemented in a commercial statistical software package [7] (Stata version 11.1, StataCorp, Texas, USA). This showed that 90.8% (215419/237,152) of the regressions for the body composition outcome possessed significant heteroskedasticity. In comparison the 237,152 regressions with the neuro-cognitive outcome were also tested for heteroskedasticity and only 1.4% (3393/237,152) of these possessed significant heteroskedasticity. We therefore concluded that the source of the heteroskedasticity must be due to differences in the measured phenotypes and undertook investigations accordingly. The neuro-cognitive phenotype was negatively skewed (skewness = −0.566; se 0.472) and the body composition phenotype was positively skewed (skewness = 0.927; se 0.501). Although this is not a significant difference it may still contribute to heteroskedasticity in the regressions. A log transformation of the body composition phenotype to counteract skewness was not observed to rectify heteroskedasticity on the residual plots. A possibly more important difference between the phenotypes is that the body composition phenotype was found to contain an outlier (as defined by Tukey [8]) whereas the neuro-cognitive outcome does not contain any outliers. The presence of outliers is known to cause heteroskedasticity, especially when the sample size is small [9] and therefore we believe that this is the most likely source of the heteroskedasticity. We tested this by removing the outlier, rerunning all 237,152 regressions and retesting for heteroskedasticity. We observed that the percentage of heteroskedastic regressions dropped from 90.8% to 25.2% and therefore we conclude that this outlier significantly contributed to heteroskedasticity in the regressions. The validity of this outlier was carefully checked and it was thought to be a genuinely large value (i.e. not due to measurement or recording error). We therefore did not consider it statistically appropriate to exclude this outlier from the analysis.

Heteroskedasticity can be accommodated in a regression model using a heteroskedasticity–consistent covariance matrix estimator [10] to calculate standard errors of regression coefficients that are robust to heteroskedasticity. This can be easily incorporated into the calculation of standard errors of regression coefficients using commercial software packages (again we used Stata version 11.1, but the methods are available in most statistical software packages including R and SPSS). P values for regression coefficients are calculated using the estimates of regression coefficients and associated standard errors. New P values were therefore calculated in a heteroskedasticity-consistent (i.e. robust) manner for the body composition outcome and the distribution of these P values is shown in Figure 5.

**Figure 5:**
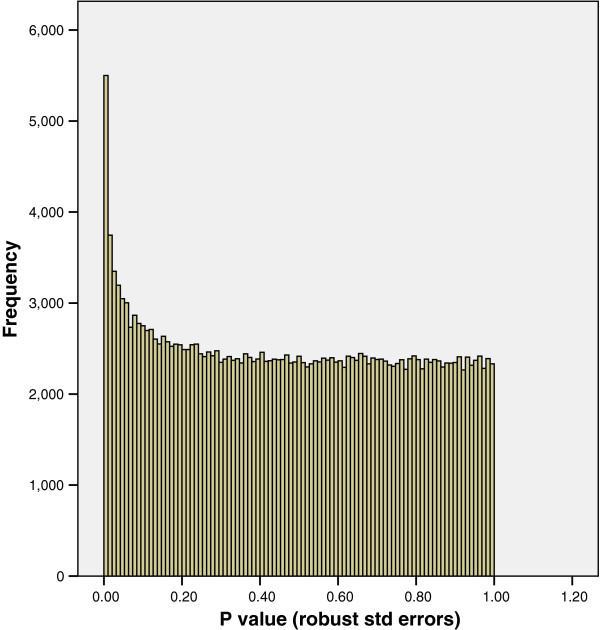
**The distribution of P values with a body composition outcome using robust standard errors.** Figure 5 indicates that a number of probes are associated with the outcome of interest as there are more P values ≤ 0.05 than would be expected by chance.

The P value distribution in Figure 5 indicates that a number of probes are associated with the outcome of interest as there are more P values ≤ 0.05 than would be expected by chance. Thus the robust estimator for the standard error of the regression coefficients leads to an acceptable distribution of P values and enables inference about which probes are likely to be significantly associated with the outcome of interest.

In order to aid interpretation of these significant associations an anonymous reviewer helpfully suggested that we calculate false discovery rates for our results. We therefore calculated Q values and hence false discovery rates using the Q-Value software of Dabney and Storey [2]. For the neuro-cognitive phenotype pi 0 (the overall proportion of null hypotheses) = 0.978. P values ≤ 0.01 had a false discovery rate of 45.3% and P values of ≤ 0.001 had a false discovery rate of 26.5%. For the body composition phenotype with P values calculated using robust estimates, pi 0 = 0.941. P values ≤ 0.01 had a false discovery rate of 42.1% and P values of ≤ 0.001 had a false discovery rate of 24.6%. It was not appropriate to calculate Q values using the classical regression P values for body composition as the required assumption of ‘null’ probes having a Uniform distribution was not met.

We were also curious as to what happened to the original P values (1058 in number) that were ≤ 0.01, represented by the first bar (far left) on Figure 3, after they had been re-estimated using robust standard errors. Figure 6 shows that the majority of these P values are still ≤ 0.01 after re-estimation. It therefore follows that the majority of significant P values calculated using heteroskedasticity-consistent standard errors were not observed to be associated when classical regression estimates were used. This therefore reinforces the principle that if statistical assumptions are not met significant associations can be missed.

**Figure 6:**
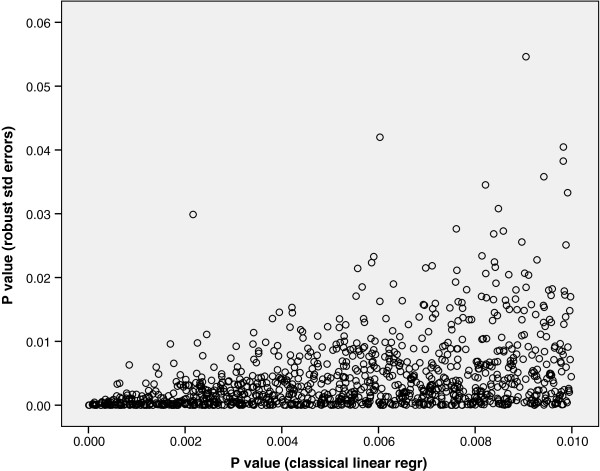
**Comparison of P values ≤ 0.01 using classical linear regression recalculated using robust standard errors.** Figure 6 shows that the majority of these P values are still ≤ 0.01 after re-estimation.

## Conclusions

A literature search for non-uniform distributions of P values shows few citations [3,5] and these both relate to statistical tests applied to expression results generated using Affymetrix microarrays. Huang et al. [5] compare gene expression profiles of tumours in three groups of mice using an Affymetrix Mouse Genechip array. When an ANOVA was performed on the expression of around 23,000 genes, a distribution of P values similar to Figure 1 was obtained. However when a *t*-test was applied to 2 of these groups, a distribution of P values similar to Figure 3 was obtained. The authors hypothesised that the reason for such a non-uniform distribution was due to excess biological similarity between some samples in the groups used for the t-test. This excess biological similarity was thought to be due to 2 pairs of samples being littermates of the same age and a further 2 pairs of samples were assayed at the same time. This resulted in the samples used for the t-test not being statistically independent. As such samples are not statistically independent this violates one of the main assumptions required for the t-test, namely that of independence i.e. observations between or within groups are not paired, dependent, correlated, or associated in any way [11]. Thus Huang et al. also observe that violation of statistical assumptions can result in an unexpected distribution of P values similar to Figure 3.

Fodor et al. [3] using Human and Mouse Affymetrix Genechip arrays, hypothesised that poorly characterised variance and normalisation strategy for a microarray can produce a non-uniform distribution of P values for null genes (genes that are not differentially expressed between groups). The authors proposed the use of a cyber t-test which uses Bayesian statistics to weight the variance of each individual gene with the variance of other genes on the array with similar intensities and also the use of an additional statistic-level normalisation to rectify the problem. They state that it is clear that one of the assumptions of the standard t-test has been violated to lead to the non-uniform distribution of P values. However if the assumptions of the t-test have not been met, a much simpler solution would be the use of non-parametric tests, such as the Mann–Whitney *U* test, which do not require an assumption of equal variance between genes.

Fodor et al. also suggested other technical reasons why a P value distribution can be non-uniform such as cross-hybridisation (probes hybridising to genes other than their intended target). Dabney and Storey [12] come to a similar conclusion when they attributed the non-uniform P value distribution of a particular analysis to errors in experimental design. This was contested by the authors [13,14] who suggested that a non-uniform distribution of P values may be a common feature of data generated by microarray experiments. However in our data technical issues to do with the array are not thought to be a problem as we obtained a plausible distribution of P values for our neuro-cognitive outcome.

The statistical analysis of ‘omics data requires careful handling, especially in the choice of statistical test. It is imperative that the assumptions behind these tests are carefully examined and any violations rectified if possible, or a more appropriate statistical test chosen (such a non-parametric test) if meaningful results are to be obtained.

## Methods

Samples analysed were from umbilical cord DNA taken from 24 participants in a cohort study. Cord DNA was selected for analysis from participants with subsequent neuro-cognitive and body composition outcomes. The phenotype measures were double entered and verified by experienced data entry staff and the data were subjected to rigorous range and consistency checking by database managers and senior statisticians. Follow up of the participants and sample collection/analysis was carried out under Institutional Review Board approval (Southampton and SW Hampshire Local Research Ethics Committee) with written informed consent obtained from parents or guardians. Investigations were conducted according to the principles expressed in the Declaration of Helsinki.

The DNA from each participant was sheared by sonication to produce fragments of between 200 and 500bp. A proportion of this DNA (input DNA) was purified and the remaining DNA incubated with His-tagged MBD2b (methyl binding domain of MeCP2) protein to form a complex which was then captured on nickel coated magnetic beads. The beads were then washed to remove unmethylated DNA fragments before DNA was eluted from the beads using an elution buffer designed to simultaneously elute methylated DNA while degrading MBD2b protein as per manufacturer’s instructions. This purified methylated DNA was then analysed using RT-PCR with primers specific for the promoter region of a housekeeping (and therefore unmethylated i.e. β-actin) gene and the ICR region of the imprinted gene H19. Methylated and input samples were labelled with the fluorescent dyes Cy3 and Cy5 respectively and then hybridised to the Agilent human promoter whole genome array (G4489A). This array contains probes (60mers) spaced every 100-300bp across the promoter regions of 17,000 of the best characterised transcripts (from -8kb to +2kb downstream of the transcription start site of each gene). The probes are arranged on two plates, one accommodating chromosomes 1 to 10 and the other accommodating chromosomes 10/11 to 22 together with X and Y. Microarray hybridisation was carried out by Oxford Gene Technology (OGT, Oxford UK) in accordance with the company’s quality control procedures using standard protocols for labelling, hybridisation and washing. Microarray slides were scanned at 5μM resolution using the extended dynamic range (High 100%, Low 10%). The slides were then feature extracted using Agilent feature extraction software 9.5.3.1. All arrays were normalised per spot and per chip using an intensity dependent normalisation (Lowess normalisation) using Genespring (http://stat-www.berkeley.edu/users/terry/zarray/Html/normspie.html). This is a within slide normalisation that adjusts for intensity dependent variation due to dye properties. The log ratio of methyl to input signal was calculated for each probe by dividing the Cy5 processed signal by Cy3 processed signal.

The following QC measures were employed for the probe log ratio values before analysis. The log ratio value for a probe was set to missing for a participant if any of the following quality control variables indicated a positive result. If either the green or red channels were saturated, were non uniform feature outliers, were background non uniform outliers, were feature population outliers or if a ‘manual flag’ variable indicated that the spot did not pass a visual check. At most 0.2% of the probe values for any participant did not pass quality control.

### Statistical methods

Statistical procedures were performed in Stata version 11.1, StataCorp, Texas, USA and SPSS version 19 (IBM, Armonk, New York).

Linear regression was performed using phenotype as the dependent variable and each standardised probe log ratio value as the predictor variable. Regression assumptions of Normality were investigated using a P-P plot. This plots the observed cumulative probability of the regression standardised residuals against the expected cumulative probability for a Normal distribution. If the regression residuals are Normally distributed the points on this plot will follow the line of equality, a straight line at a 45 degree angle passing through the coordinates (0,0), (0.5,0.5) etc. Assumptions of constant variance of the regression residuals were investigated using a plot of standardised regression residuals versus predicted values. If this plot shows that the variance of the regression residuals does not increase with the predicted value the assumption of homoskedasticity (constant variance) has not been violated. However if the plot shows the regression residuals increasing with predicted value (as in Figure 4), this indicates heteroskedasticity.

Heteroskedasticity was tested with the Cook-Weisberg test [6] using regression residuals implemented in Stata version 11.1, this is a score test which tests the hypothesis that the variance of the regression residuals is constant. Regressions were then rerun using a heteroskedasticity–consistent covariance matrix estimator proposed by White [10], to recalculate the standard errors of regression coefficients. Subsequently, new P values for the regression coefficients were calculated using the original estimate of the regression coefficient and the heteroskedasticity–consistent standard errors for each probe.

## Competing interests

SJB declares that she has no financial or non-financial competing interests. SRC declares that she has no financial or non-financial competing interests. KAL declares that she has no financial or non-financial competing interests. KMG has acted as a consultant to Abbott Nutrition and Nestle Nutrition, and has received reimbursement for speaking at an Abbott Nutrition Conference on Pregnancy Nutrition and Later Health Outcomes and at a Nestle Nutrition Institute Workshop. He is part of an academic consortium that has received research funding from Abbott Nutrition, Nestec and Danone. HMI declares that she has no financial or non-financial competing interests.

## Authors’ contributions

SJB conceived of the study, performed the statistical analysis and drafted the manuscript. SRC provided expertise in statistical programming and assisted with the statistical analysis for the study KAL supervised the laboratory work, provided expertise in biology and helped to draft the Materials and methods section. KMG provided expertise in biology and drafted the Materials and methods section. HMI provided statistical input and expertise. All authors reviewed and edited the manuscript. All authors read and approved the final manuscript.
